# A paleolithic diet is more satiating per calorie than a mediterranean-like diet in individuals with ischemic heart disease

**DOI:** 10.1186/1743-7075-7-85

**Published:** 2010-11-30

**Authors:** Tommy Jönsson, Yvonne Granfeldt, Charlotte Erlanson-Albertsson, Bo Ahrén, Staffan Lindeberg

**Affiliations:** 1 Department of Clinical Science, B11 BMC, University of Lund, SE-221 84 Lund, Sweden; 2 Department of Food Technology, Engineering and Nutrition, University of Lund, Lund, Sweden; 3 Section of Metabolism, Endocrinology and Diabetes, Department of Experimental Medicine, University of Lund, Lund, Sweden

## Abstract

**Background:**

We found marked improvement of glucose tolerance and lower dietary energy intake in ischemic heart disease (IHD) patients after advice to follow a Paleolithic diet, as compared to a Mediterranean-like diet. We now report findings on subjective ratings of satiety at meals and data on the satiety hormone leptin and the soluble leptin receptor from the same study.

**Methods:**

Twenty-nine male IHD patients with impaired glucose tolerance or diabetes type 2, and waist circumference > 94 cm, were randomized to *ad libitum *consumption of a Paleolithic diet (n = 14) based on lean meat, fish, fruit, vegetables, root vegetables, eggs, and nuts, or a Mediterranean-like diet (n = 15) based on whole grains, low-fat dairy products, vegetables, fruit, fish, and oils and margarines during 12 weeks. In parallel with a four day weighed food record the participants recorded their subjective rating of satiety. Satiety Quotients were calculated, as the intra-meal quotient of change in satiety during meal and consumed energy or weight of food and drink for that specific meal. Leptin and leptin receptor was measured at baseline and after 6 and 12 weeks. Free leptin index was calculated as the ratio leptin/leptin receptor.

**Results:**

The Paleolithic group were as satiated as the Mediterranean group but consumed less energy per day (5.8 MJ/day vs. 7.6 MJ/day, Paleolithic vs. Mediterranean, p = 0.04). Consequently, the quotients of mean change in satiety during meal and mean consumed energy from food and drink were higher in the Paleolithic group (p = 0.03). Also, there was a strong trend for greater Satiety Quotient for energy in the Paleolithic group (p = 0.057). Leptin decreased by 31% in the Paleolithic group and by 18% in the Mediterranean group with a trend for greater relative decrease of leptin in the Paleolithic group. Relative changes in leptin and changes in weight and waist circumference correlated significantly in the Paleolithic group (p < 0.001) but not in the Mediterranean group. Changes in leptin receptor and free leptin index were not significant.

**Conclusions:**

A Paleolithic diet is more satiating per calorie than a Mediterranean-like diet.

**Trial registration:**

ClinicalTrials.gov NCT00419497

## Background

We found marked improvement of glucose tolerance in ischemic heart disease (IHD) patients with impaired glucose tolerance or diabetes type 2 after advice to follow a Paleolithic diet, as compared to a Mediterranean-like diet [1]. To our knowledge, this was the first randomized, controlled study on the health effects of a Paleolithic diet. The Paleolithic diet was based on lean meat, fish, fruits, vegetables, root vegetables, eggs and nuts. Control subjects, who were advised to follow a Mediterranean-like diet based on whole grains, low-fat dairy products, fish, fruit and vegetables, did not significantly improve their glucose tolerance despite significant decreases of weight and waist circumference. The main differences in food consumption, as reported in four day weighed food records, were a much lower intake of cereals and dairy products, a higher intake of fruit and nuts and a trend for higher intake of vegetables in the Paleolithic group compared to the Mediterranean group [1]. After publication of our study, a systematic review on the evidence of a causal link between Mediterranean diet and cardiovascular disease found strong evidence for a protective effect of vegetables, nuts and monounsaturated fat on coronary heart disease, whereas the evidence for whole grain was moderate and for milk products weak [2]. This review, together with the differences we found between Paleolithic and Mediterranean diet, is further evidence for a specific role of the Paleolithic diet on protection of the heart. The more pronounced improvement of glucose tolerance in the Paleolithic group was independent of similar weight loss in both groups (-5.0 kg vs. -3.8 kg, Paleolithic vs. Mediterranean) and a greater decrease in waist circumference (-5.6 cm and -2.9 cm, Paleolithic vs. Mediterranean) and lower reported energy intake in the Paleolithic group (5.6 MJ/day vs. 7.5 MJ/day, Paleolithic vs. Mediterranean) [1].

Thus, the individuals in the Paleolithic group reportedly consumed less energy compared to the Mediterranean group, but were they as satiated? The lower energy intake in the Paleolithic group could be due to either of two scenarios when it comes to satiety. In the first scenario, there would be a difference in subjective satiety between the groups, such that the subjects in the Paleolithic group were hungrier but for some reason chose not to eat more, despite that no restrictions on energy intake were given (to either group). This could indicate dieting with a conscious intent to eat fewer calories on the Paleolithic diet, or perhaps the Paleolithic diet was simply perceived as less palatable and the subjects chose to go a bit hungrier rather than eating more. In the second scenario, there would be no difference in subjective satiety between groups, suggesting that the Paleolithic diet was more satiating per energy unit than the Mediterranean-like diet. This would be an important finding, since a diet which satiates more per energy unit could be helpful in preventing or treating overweight and obesity and associated diseases. Having thus demonstrated a greater satiating capacity of a Paleolithic diet, what could the dietary components be that account for this capacity? It has been suggested that a Paleolithic diet could be more satiating due to macronutrient composition and fiber content [3,4]. Another possible explanation is that dietary components specific to an agricultural diet cause leptin resistance with ensuing disturbance of appetite regulation [5]. To address these questions on satiety and its dietary mechanisms, we now report further findings on subjective ratings of satiety and data on the satiety hormone leptin and the soluble leptin receptor from the same population and material described in our study above [1].

### The concept of satiation and its determinants

Foods differ in their satiating capacity, partly due to their nutritional composition [6,7]. The impact of foods on subjectively perceived measures of motivation to eat (e.g. hunger, fullness) can be quantified by fixed point (category) scales and visual analogue scales [6,8]. Participants in a trial assess their motivation to eat and mark this on a graded scale. Subjective ratings of appetite usually show positive correlations with the amount of food consumed, and can be considered a valid indicator of the strength of appetite [6-8]. The satiating effect of different foods has been frequently assessed by the Satiety Quotient, which gives a measure of the extent to which the food eaten reduces subjective appetite per unit of intake (e.g., per kg or MJ) for that specific meal and is predictive of energy intake [6]. The Satiety Quotient is calculated by the following formula:

$$\begin{array}{cc} & \text{satiety rating pre-eating episode}-\\ & \text{satiety rating post-eating episode}\\ \text{Satiety Quotient }= & --------------------\\ & \text{food intake of eating episode} \end{array}$$

### Leptin, the leptin receptor and free leptin index

Leptin is a peptide hormone, mainly secreted from adipose tissue, which influences appetite, reproduction, hematopoiesis, angiogenesis, blood pressure, bone mass, energy homeostasis, and immune and neuroendocrine function (for review see [9]). Circulating leptin levels signal to the brain how much energy is stored and how much food has been consumed [10], and an increased leptin level in rodents and humans results in decreased food intake and increased energy expenditure [11]. Since obese humans show elevated levels of circulating leptin, and obtain limited weight loss from leptin treatment, many researchers consider obese humans to be leptin resistant [9,11]. The homeostatic response to involuntary overfeeding suggests that leptin resistance could be a cause rather than a consequence of obesity [12]. Leptin circulates in both free and protein-bound forms, and the soluble leptin receptor (SLR) is the major binding component of leptin in plasma and crucial for leptin action [13,14]. Leptin correlates significantly with body mass index, while SLR is inversely correlated with body mass index[15]. In lean subjects, there is a molar equivalence of free leptin to SLR, whereas in morbidly obese subjects a 25-fold excess of free leptin has been reported [15,16]. It has been suggested that hyperleptinemia, low SLR levels and a low fraction of leptin bound to SLR are all markers of leptin resistance and associated with the metabolic syndrome [17-19]. Free leptin index is calculated as the ratio between levels of circulating leptin and SLR [20], and correlates in healthy humans positively with body fat mass, plasma insulin and masked hypertension, and negatively with waist-hip ratio [21-23].

## Methods

### Population

The study was a 12-week controlled dietary intervention trial in 29 (out of 38 eligible) male IHD patients with waist circumference >94 cm and increased blood glucose at screening oral glucose tolerance test (OGTT) with capillary blood glucose measured fasting and at 2 hours, or known diabetes type 2, recruited from the Coronary Care Unit at Lund University Hospital, Sweden. Standard methods were used for glucose testing and definitions of glucose tolerance [1]. We included patients with any of the following conditions: an ongoing acute coronary syndrome, a history of myocardial infarction diagnosed by creatinine kinase MB isoenzyme or troponin elevation, percutaneous coronary intervention or coronary artery bypass graft surgery or angiographically diagnosed coronary stenosis ≥30%. Exclusion criteria were body mass index (BMI) <20 kg/m^2^, serum creatinine >130 μmol/L, poor general condition, dementia, unwillingness/inability to prepare food at home, participation in another medical trial, chronic inflammatory bowel disease, type 1 diabetes and drug treatment with hypoglycemic agents, warfarin or oral steroids. Other drugs were not restricted, and treatment with statins and beta blockers were usually initiated and/or changed during the trial. In addition to the 29 patients who completed the trial, nine randomized subjects were excluded for the following reasons: worsening general condition (two in each group), non-willingness to continue (n = 3, all in the Paleolithic group) or missing OGTT data (one in each group). Approval of the study was obtained from the Medical Ethics Committee at Lund University, and all individuals gave written informed consent to participate in the study.

### Intervention

All eligible subjects were informed of the intention to compare two diets and that it was unknown if any of them would be superior to the other with regard to weight reduction and improved glucose metabolism. Subjects were randomized to one of two diets: a Mediterranean-like diet (n = 15) or a Paleolithic diet (n = 14). All subjects were informed individually (by SL or one of two registered nurses with special nutrition education (the same in the two groups)) during two one-hour sessions and were given written dietary advice and many food recipes. The Mediterranean-like diet was based on whole-grain cereals, low-fat dairy products, potatoes, legumes, vegetables, fruit, fatty fish, and refined fats rich in monounsaturated fatty acids and alpha-linolenic acid. Only subjects in the Mediterranean group were informed of the possible benefits of Mediterranean-like diets rich in whole grains and about the Lyon Diet Heart Study [24]. The Mediterranean group was also educated by use of a dietary questionnaire for nutrition counseling ('20 questions') used in a successful health promotion program, 'Live For Life', which led to lowered cardiovascular and total mortality in the Habo municipality, Sweden [25]. For details on questionnaire, see [1].

Only subjects in the Paleolithic group were educated in the concept of evolutionary health promotion [26] and the potential benefits of a Paleolithic diet. They were advised to increase their intake of lean meat, fish, fruit and vegetables and to avoid all kinds of dairy products, cereals (including rice), beans, sugar, bakery products, soft drinks and beer. The following items were accepted in limited amounts for the Paleolithic group: eggs (one or fewer per day), nuts (preferentially walnuts), potatoes (two or fewer medium-sized per day), rapeseed or olive oil (one or fewer tablespoons per day). The intake of other foods was not restricted and no advice was given with regard to proportions of food categories (e.g. animal vs. plant foods). The type of dietary advice given to Mediterranean subjects was similar to the established program at the coronary care unit. Since the required increase in education intensity in order to match the Paleolithic group was rather small, no 'usual care' control group was considered necessary. Advice about regular physical activity was given equally to the two groups. Both groups were advised not to consume more than one glass of wine per day.

### Outcome measures

**Figure 1:**
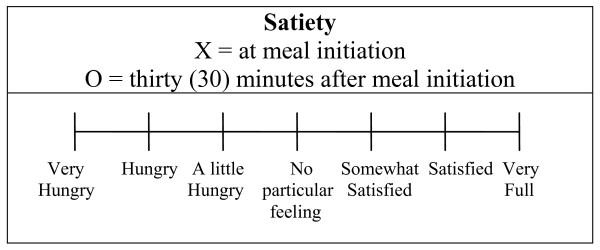
**Rating scale used to assess subjective satiety (modified from Holt et al 1992)**. In parallel with a four day weighed food record the participants also recorded their subjective rating of satiation at meal initiation and 30 minutes after meal initiation on a 7-point equal interval, bipolar scale of hunger/fullness modified after Holt et al 1992. This scale was anchored at -3 ("Very Hungry") with a midpoint at 0 ("No particular feeling") through + 3 ("Very Full"). The scale yields numeric results in units termed Rating Scale units (RS). The participants were encouraged to record their subjective rating of satiation between marked intervals if necessary, and this way of recording was common. The recorded subjective satiation was then assessed by TJ to the first decimal. For example, a recorded subjective satiation halfway between "Satisfied" and "Very Full" would yield the result 2.5 Rating Scale units.

A four day weighed food record on four consecutive days, including one weekend day, was recorded by the participants, starting 15 ± 5 days after initiating the dietary change. Participants weighed each food item on a digital weighing scale (that could be set to zero) lent by the study. In our previous report from this study, we calculated dietary nutrients using Matsedel dietary analysis software (Kost och Näringsdata AB, Bromma, Sweden) [1]. To obtain more information on dietary nutrients, and to obtain similar information as in our latest study on Paleolithic diet in subjects with diabetes [27], YG recalculated nutrient compositions in this study using data from The Swedish Food Database of the National Food Administration in Sweden. GL and GI for the two diets were calculated. The underlying concept of dietary GL and dietary GI is food GI, introduced by Jenkins et al [28], reflecting the postprandial glucose response after a specific food rich in carbohydrate, and expressing the quality of the carbohydrates. Wolever and Jenkins also suggested the possibility of ranking diets based on dietary GI calculated from the proportional GI contribution of the included foods containing carbohydrate [29]. To include also the quantity of carbohydrates consumed GL was introduced by Salmerón et al expressing the glycemic effect of the diet [30]. While dietary GI is expressing the quality of the carbohydrates consumed GL represent both the quantity and the quality of the carbohydrates consumed. Thus, dietary GL in this study was calculated as the result from multiplying available carbohydrate (g) for the food reported by the subjects during the 4-day weighed food record with the specific food's GI divided by 100. Available carbohydrate was based on total carbohydrate minus dietary fibre. The food's GI values (using glucose as reference) were taken from the compilation by Foster-Powell et al [31]. Dietary GI was calculated as 100 multiplied with dietary GL divided by the amount of available carbohydrate (g) in the diet. In parallel with this four day weighed food record the participants also recorded the time for each meal including snacks. They also recorded their subjective rating of satiation at meal initiation and 30 minutes after meal initiation on a 7-point equal interval, bipolar scale of hunger/fullness modified after Holt et al 1992 (Figure 1) [32]. This scale was anchored at -3 ("Very Hungry") with a midpoint at 0 ("No particular feeling") through + 3 ("Very Full"). The scale yields numeric results in units termed Rating Scale units (RS). The participants were encouraged to record their subjective rating of satiation between marked intervals if necessary, and this way of recording was common. The recorded subjective satiation was then assessed by TJ to the first decimal. For example, a recorded subjective satiation halfway between "Satisfied" and "Very Full" would yield the result 2.5 Rating Scale units. This scale was used since it had been assessed as reasonably sensitive and reliable compared to similar scales, and the measurement at thirty minutes was chosen for convenience of the study subjects, since this had been assessed as being as predictive of the satiety value of a given food as a sixty minutes testing period [8]. Change in satiety during meal was calculated as change in satiety between meal initiation and 30 minutes after meal initiation. Quotients of mean change in satiety during meal and mean consumed energy or weight of food and drink per meal were calculated. Also, Satiety Quotients were calculated, as the intra-meal quotient of change in satiety during meal and consumed energy or weight of food and drink for that specific meal. Fasting plasma samples were taken before 9.00 a.m. at baseline and after 6 weeks and 12 weeks, and were analyzed for leptin and leptin receptor. Serum leptin analysis was measured by a commercially available RIA (Human Leptin RIA kit, Linco Research Inc., St. Charles, MO), and serum leptin receptor was measured by a commercially available ELISA (RD194002100 BioVendor Laboratory Medicine, Inc., Brno, Czech Republic). The free leptin index was calculated as the ratio of leptin to leptin receptor. Body weight, waist circumference and serum lipids were measured by use of standard methods as described in [1].

### Statistical analysis

Assignment of patients to the two groups was made by use of minimization, a restricted randomization procedure which lowers the risk of group differences at baseline [33], using capillary blood glucose levels at screening (Diabetes: No/Yes) and BMI (below or above 27) as restricting variables. A two-way paired *t *test was used to analyze within-subject changes in absolute and relative values, while a two-way unpaired *t *test and repeated-measures ANOVA were used to analyze between-subject differences in these changes. Bivariate correlation and linear regression was used for post hoc analysis. Continuous variables showed reasonable normal distribution in normal plots. P < 0.05 was chosen for statistical significance. Data and results are expressed as mean ± standard deviation.

## Results

The two groups differed at baseline only with regard to age being higher (p = 0.01) in the Paleolithic group [1]. There was no relationship between age and any of the outcome variables at study start. Our previously reported marked improvement of glucose tolerance in the Paleolithic group was not correlated to changes in levels of satiety, leptin, leptin receptor or free leptin index. There was no significant difference between groups in measures of subjective satiety at meal initiation and 30 minutes after meal initiation or in change in satiety during meal (Table 1). There was also no difference between groups in length of time between meals or number of meals per day (Table 1). Recalculation of food nutrient composition confirmed our previous finding that the Paleolithic group consumed significantly less energy per day than the Mediterranean group (5.8 ± 2.6 MJ/day vs. 7.6 ± 1.2 MJ/day, Paleolithic vs. Mediterranean, p = 0.04, Table 1) with no difference between groups in consumption of food in terms of weight per day (1493 ± 607 g/day vs. 1649 ± 273 g/day, Paleolithic vs. Mediterranean, p = 0.4, Table 1). Consequently, there was a trend for consuming food with significantly lower energy density in the Paleolithic group (4.5 ± 1.4 kJ/g vs. 5.4 ± 1.0 kJ/g, Paleolithic vs. Mediterranean, p = 0.07, Table 2). Also, the quotients of mean change in satiety during meal and mean consumed energy from food and drink were higher in the Paleolithic group (2.5 ± 1.3 RS/MJ vs. 1.6 ± 0.5 RS/MJ, Paleolithic vs. Mediterranean, p = 0.03, Table 1), and there was a strong trend for greater Satiety Quotient for energy in the Paleolithic group (2.7 ± 1.4 RS/MJ vs. 1.8 ± 0.7 RS/MJ, Paleolithic vs. Mediterranean, p = 0.057, Table 1). There was no difference between groups in quotients of mean change in satiety during meal and mean consumed weight from food and drink or in Satiety Quotient for weight (Table 1). One individual in the Paleolithic group was an outlier in terms of change in satiety during meal, with values more than two standard deviations below both the Paleolithic and Mediterranean group mean. Without the outlier, the strong trend for higher Satiety Quotient for energy in the Paleolithic group becomes significant (2.8 ± 1.3 RS/MJ vs. 1.8 ± 0.7 RS/MJ, Paleolithic vs. Mediterranean, p = 0.02). Excluding the outlier does not change any other group comparisons in satiety.

**Table 1:** Effect of Paleolithic diet compared to Mediterranean diet on individual mean measures of satiety (group mean ± SD)

	Paleolithic diet	Mediterranean diet	P*
	(n = 13)	(n = 14)	
Time between meals (hours:minutes)	03:21 ± 00:58	03:25 ± 00:48	0,8
Meals per day	4,7 ± 0,9	4,5 ± 0,9	0,6
Energy from food and drink per meal (MJ)	1,2 ± 0,6	1,7 ± 0,4	0,02
Energy from food and drink per day (MJ)	5,8 ± 2,6	7,6 ± 1,2	0,04
Weight of food and drink per meal (g)**	315 ± 132	372 ± 83	0,2
Weight of food and drink per day (g)**	1493 ± 607	1649 ± 273	0,4
Satiety at meal initiation (RS)	-1,0 ± 0,8	-1,0 ± 0,5	1,0
Satiety 30 minutes after meal initiation (RS)	1,6 ± 0,7	1,7 ± 0,3	0,7
Change in satiety during meal (RS)	2,6 ± 1,0	2,6 ± 0,6	0,9
Quotient of mean change in satiety during meal and mean weight of food and drink per meal (RS/kg)**	9,9 ± 5,6	7,3 ± 1,6	0,12
Quotient of mean change in satiety during meal and mean energy from food and drink per meal (RS/MJ)	2,5 ± 1,3	1,6 ± 0,5	0,03
Satiety Quotient for weight (RS/kg)**	11,3 ± 6,8	9,9 ± 4,9	0,5
Satiety Quotient for energy (RS/MJ)	2,7 ± 1,4	1,8 ± 0,7	0,057

Satiety estimated with rating scale used to assess subjective satiety from 4 day weighed food records started 15 ± 5 days after initiating dietary change. *P for difference between diets in a two-sided t-test with independent samples. **Excluding weight of table water. Measures of subjective satiety in Rating Scale units (RS). Satiety Quotient is the intra-meal quotient of change in satiety during meal and consumed energy or weight of food and drink for that specific meal.

**Table 2:** Average food eaten per day during the Paleolithic and Mediterranean diet (mean ± SD)

		Paleolithic diet	Mediterranean diet	P*
		(n = 14)	(n = 13)	
Total weight** (g)		1493 ± 607	1649 ± 273	0,4
Total energy	(MJ)	5,8 ± 2,6	7,6 ± 1,2	0,04
	(kcal)	1388 ± 629	1823 ± 295	0,04
Energy density (kJ/g)**		4,5 ± 1,4	5,4 ± 1,0	0,07
Protein	(g)	92 ± 46	88 ± 17	0,8
	(E%)	27 ± 6	20 ± 3	0,002
Carbohydrate	(g)	129 ± 58	211 ± 37	0,0002
	(E%)	39 ± 11	47 ± 7	0,02
Fat	(g)	46 ± 26	59 ± 18	0,13
	(E%)	28 ± 7	28 ± 6	0,9
Alcohol	(g)	6 ± 7	5 ± 6	0,6
	(E%)	3 ± 4	2 ± 2	0,3
Fiber	(g)	22 ± 14	27 ± 6	0,2
	(E%)	3 ± 1	3 ± 1	0,9
Glycemic Load (g)		63 ± 29	110 ± 22	0,0001
Dietary Glycemic Index		49 ± 5	51 ± 4	0,3
Monosaccharides (g)		50 ± 32	37 ± 17	0,2
Disaccharides(g)		32 ± 16	40 ± 15	0,2
Sucrose (g)		29 ± 16	23 ± 12	0,3
Saturated fatty acid (g)		13 ± 6	19 ± 6	0,01
Monounsaturated fatty acid(g)		18 ± 9	22 ± 8	0,2
Polyunsaturated fatty acid (g)		10 ± 8	12 ± 6	0,5
Fatty acid C4:0-C10:0 (g)		0,1 ± 0,2	1,1 ± 0,6	0,00004
Fatty acid C12:0 (g)		0,2 ± 0,3	0,8 ± 0,5	0,001
Fatty acid C14:0 (g)		1,0 ± 0,6	1,9 ± 0,8	0,003
Fatty acid C16:0 (g)		8 ± 4	11 ± 3	0,06
Fatty acid C16:1 (g)		1,6 ± 1,4	1,4 ± 0,7	0,6
Fatty acid C18:0 (g)		3,0 ± 1,6	3,8 ± 1,1	0,2
Fatty acid C18:1, oljesyra (g)		15 ± 7	18 ± 6	0,2
Fatty acid C18:2, n-6, Linoleic acid (g)		6 ± 4	8 ± 4	0,11
Fatty acid C18:3, n-3, ALA (g)		1,2 ± 1,1	1,5 ± 0,8	0,6
Fatty acid C20:0 (g)		0,03 ± 0,03	0,05 ± 0,04	0,07
Fatty acid C20:4, n-6 (g)		0,2 ± 0,2	0,1 ± 0,1	0,12
Fatty acid C20:5, n-3, EPA (g)		0,7 ± 0,7	0,5 ± 0,5	0,6
Fatty acid C22:5, n-3 (g)		0,2 ± 0,3	0,1 ± 0,1	0,4
Fatty acid C22:6, n-3, DHA (g)		1,5 ± 1,7	1,1 ± 0,9	0,5
Cholesterol (mg)		402 ± 224	287 ± 129	0,11
Vitamin A, Retinolequivalents (μg)		766 ± 388	747 ± 359	0,9
Vitamin A, Retinol (μg)		255 ± 218	447 ± 250	0,04
Vitamin A, Caroten (μg)		5288 ± 4365	2891 ± 2072	0,09
Vitamin D (μg)		13 ± 15	9 ± 5	0,4
Vitamin E (mg)		10 ± 5	10 ± 3	0,9
Vitamin E, Alpha-tocopherol (mg)		10 ± 5	10 ± 3	0,9
Vitamin B-1, Thiamin (mg)		1,5 ± 1,0	1,5 ± 0,4	0,9
Vitamin B-2, Riboflavin (mg)		1,4 ± 0,6	1,8 ± 0,6	0,13
Vitamin B-6 (mg)		3,6 ± 2,5	2,5 ± 0,6	0,2
Vitamin B-12 (μg)		9,2 ± 7,5	7,2 ± 3,6	0,4
Vitamin B, Folate (μg)		418 ± 335	280 ± 112	0,2
Vitamin C, Ascorbic acid (mg)		253 ± 227	126 ± 83	0,08
Niacinequivalents (mg)		46 ± 25	39 ± 9	0,4
Niacin (mg)		29 ± 16	23 ± 7	0,2
Phosphorus (mg)		1156 ± 568	1465 ± 195	0,08
Iron (mg)		11 ± 4	12 ± 2	0,4
Potassium (mg)		3889 ± 1951	3402 ± 578	0,4
Calcium (mg)		374 ± 206	772 ± 224	0,0001
Magnesium (mg)		310 ± 310	342 ± 56	0,5
Sodium (mg)		1497 ± 416	3140 ± 758	0,000001
Selenium (μg)		77 ± 53	64 ± 27	0,4
Zinc (mg)		10 ± 4	11 ± 2	0,5
Ash (g)		15 ± 6	19 ± 3	0,06
Water from food (g)		1052 ± 476	864 ± 192	0,2
Fruits (g)		513 ± 350	262 ± 171	0,03
Vegetables (g)		368 ± 299	198 ± 79	0,07
Potatoes (g)		68 ± 50	87 ± 80	0,5
Nuts (g)		10 ± 12	1 ± 3	0,02
Meat (g)		194 ± 106	70 ± 54	0,001
Meat products (g)		67 ± 100	82 ± 66	0,6
Fish (g)		114 ± 93	74 ± 49	0,2
Eggs (g)		33 ± 38	22 ± 24	0,3
Beans (g)		3 ± 12	20 ± 34	0,09
Cereals without rice (g)		21 ± 50	257 ± 88	0,00000001
Rice (g)		0 ± 0	20 ± 27	0,01
Milk/milk products (g)		39 ± 102	308 ± 171	0,00005
Oil (g)		0,0 ± 0,0	1,3 ± 2,8	0,10
Sauce (g)		1 ± 5	34 ± 67	0,09
Bakery (g)		3 ± 8	9 ± 23	0,4
Jam (g)		1 ± 3	4 ± 9	0,2
Spirits (g)		0,0 ± 0,0	1,4 ± 5,1	0,3
Wine (g)		62 ± 67	37 ± 51	0,3
Beer (g)		11 ± 27	29 ± 55	0,3
Sweet beverages (g)		1 ± 2	45 ± 103	0,13
Juice (g)		37 ± 72	82 ± 135	0,3
Table water (g)		206 ± 293	354 ± 677	0,5
Coffee (g)		272 ± 215	441 ± 387	0,2
Tea (g)		125 ± 246	87 ± 142	0,6

Estimated from 4 day weighed food records. *P for difference between diets in a two-sided t-test with dependent samples. **Excluding weight of non-energy containing beverage such as table water, coffee and tea. E% = percent energy from total macronutrient energy.

During the 12-week dietary intervention leptin decreased significantly by 31% in the Paleolithic group (p = 0.0006) and by 18% in the Mediterranean group (p = 0.03) (Table 3). There was a trend for greater relative decrease of leptin in the Paleolithic group compared to the Mediterranean group (p = 0.15, Table 3). After 12 weeks, leptin receptor concentration had increased by 17% in the Paleolithic group and by 33% in the Mediterranean group with no significant difference between groups (Table 3). Free leptin index decreased by 28% in the Paleolithic group and by 30% in the Mediterranean group with no significant difference between groups after 12 weeks (Table 3). Comparisons between groups in absolute and relative changes of leptin, the leptin receptor and free leptin index were also non-significant in repeated measurements ANOVA (data not shown).

**Table 3:** Effect of Paleolithic diet compared to Mediterranean diet on levels of leptin, leptin receptor and free leptin index (mean ± SD)

	Paleolithic diet	Mediterranean diet	P*
	(n = 14)	(n = 15)	
*Fasting plasma leptin, ng/ml*			
Baseline	10,7 ± 3,9	13,5 ± 11,0	0,4
6 weeks	6,6 ± 3,0	10,9 ± 8,5	0,08
12 weeks	7,1 ± 3,2	11,0 ± 8,4	0,11
Change 0-6 weeks	-4,0 ± 3,1	-2,6 ± 3,3	0,2
P for change within groups 0-6 weeks	0,0003	0,01	
Relative change 0-6 weeks,%	-34 ± 25	-19 ± 20	0,08
Relative change 0-6 weeks, **outlier excluded,%	-37 ± 23	-19 ± 20	0,03
Change 0-12 weeks	-3,6 ± 3,0	-2,5 ± 4,0	0,4
P for change within groups 0-12 weeks	0,0006	0,03	
Relative change 0-12 weeks,%	-31 ± 26	-18 ± 22	0,15
Relative change 0-12 weeks, **outlier excluded,%	-35 ± 21	-18 ± 22	0,04
*Fasting plasma leptin receptor, ng/ml*			
Baseline	19,0 ± 8,9	14,9 ± 5,7	0,14
6 weeks	20,5 ± 12,3	19,6 ± 8,7	0,8
12 weeks	20,6 ± 10,8	18,1 ± 5,7	0,5
Change 0-6 weeks	1,5 ± 7,2	4,7 ± 8,2	0,3
P for change within groups 0-6 weeks	0,5	0,04	
Relative change 0-6 weeks,%	13 ± 45	37 ± 50	0,2
Change 0-12 weeks	1,5 ± 7,0	3,2 ± 7,2	0,5
P for change within groups 0-12 weeks	0,4	0,10	
Relative change 0-12 weeks,%	17 ± 51	33 ± 49	0,4
*Free leptin index*			
Baseline	0,7 ± 0,5	1,1 ± 1,3	0,3
6 weeks	0,5 ± 0,5	0,7 ± 0,7	0,3
12 weeks	0,5 ± 0,4	0,6 ± 0,4	0,3
Change 0-6 weeks	-0,3 ± 0,6	-0,4 ± 0,7	0,5
P for change within groups 0-6 weeks	0,09	0,04	
Relative change 0-6 weeks,%	-29 ± 47	-33 ± 29	0,8
Change 0-12 weeks	-0,2 ± 0,5	-0,5 ± 1,0	0,4
P for change within groups 0-12 weeks	0,08	0,08	
Relative change 0-12 weeks,%	-28 ± 43	-30 ± 33	0,9

*P for difference between groups in a two-sided t-test with independent samples. ** One individual from the Paleolithic group was an outlier in terms of cereal intake.

**Figure 2:**
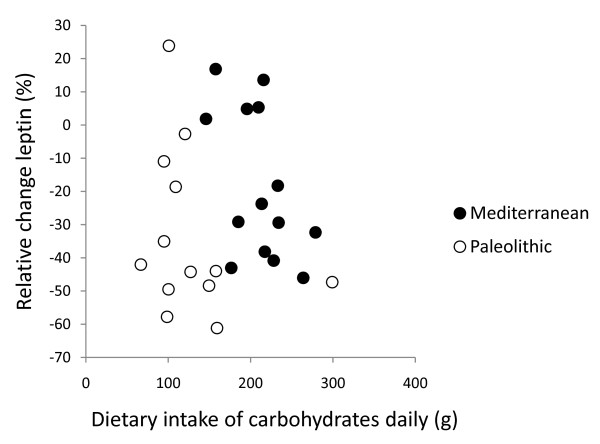
**Individual values of relative change in leptin versus dietary intake of carbohydrates daily**. The figure show individual values of relative change in leptin after 12 weeks versus dietary intake of carbohydrates daily. Individuals from the Paleolithic group are depicted with open circles (○) and individuals from the Mediterranean group with closed circles (●).

**Figure 3:**
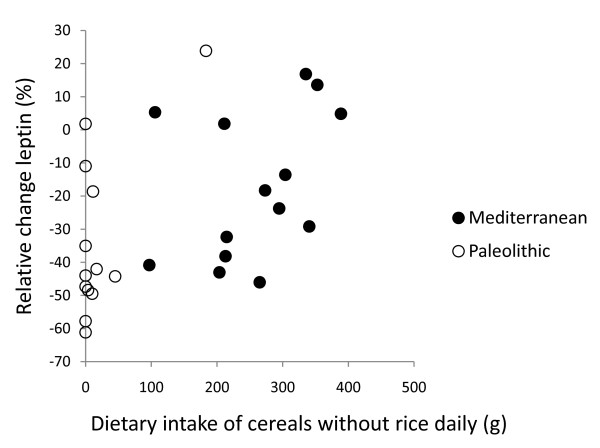
**Individual values of relative change in leptin versus dietary intake of cereals without rice daily**. The figure show individual values of relative change in leptin after 12 weeks versus dietary intake of cereals without rice daily. Individuals from the Paleolithic group are depicted with open circles (○) and individuals from the Mediterranean group with closed circles (●).

**Figure 4:**
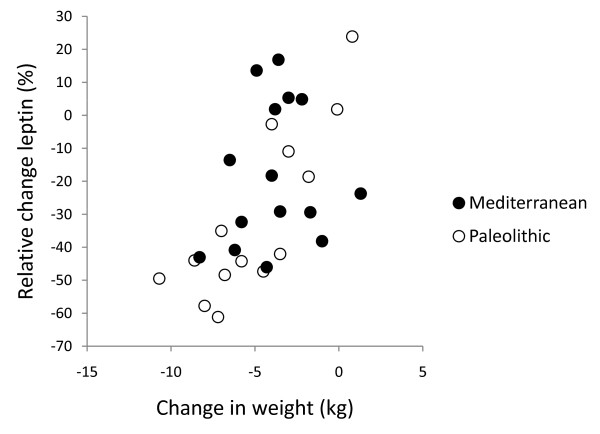
**Individual values of relative change in leptin versus change in weight**. The figure show individual values of relative change in leptin after 12 weeks versus change in weight. Individuals from the Paleolithic group are depicted with open circles (○) and individuals from the Mediterranean group with closed circles (●).

**Figure 5:**
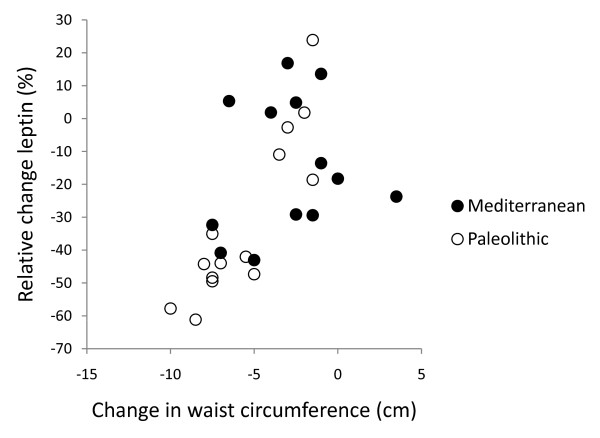
**Individual values of relative change in leptin versus change in waist circumference**. The figure show individual values of relative change in leptin after 12 weeks versus change in waist circumference. Individuals from the Paleolithic group are depicted with open circles (○) and individuals from the Mediterranean group with closed circles (●).

In post hoc analysis, the strongest correlation between relative change in leptin after 12 weeks and dietary variables was with intake of cereals excluding rice (Pearson correlation 0.50, p = 0.008, Figure 2, 3, 4 and 5). Furthermore, one subject in the Paleolithic group consumed 183 g cereals without rice per day, which was well within the variation for the Mediterranean group (257 ± 88 g/day, mean ± SD), but more than three standard deviations above the Paleolithic group (21 ± 50 g/day, mean ± SD). The Paleolithic individual is thus clearly an outlier in terms of cereal consumption for the Paleolithic group, but normal in terms of cereal consumption for the Mediterranean group. When this Paleolithic outlier is excluded, the trend for difference between groups in relative leptin change during the study becomes significant (-35 ± 21% vs. -18 ± 22%, Paleolithic vs. Mediterranean, p = 0.04, Table 3).

After 12 weeks, relative changes in leptin correlated significantly with changes in weight and waist circumference (p < 0.001 for both) in the Paleolithic group, but there was no such correlation in the Mediterranean group (Figure 4 and 5). After 12 weeks, relative changes in free leptin index also correlated significantly with changes in waist circumference (p = 0.04) but not with changes in weight in the Paleolithic group, and there was no correlation with either in the Mediterranean group. The correlation between cereal intake without rice and relative change in leptin remained significant when changes in weight were controlled for, but not when changes in waist were controlled for (data not shown).

Reported food consumption differed between the two groups such that subjects in the Paleolithic group had a much lower intake of cereals and milk, and a higher intake of fruit, nuts and meat and also a trend for higher intake of vegetables (Table 2). Absolute intake of protein did not differ between groups, but relative intake of protein (as a percentage of total macronutrient energy intake [E%]) was higher in the Paleolithic group (27 ± 6 E% vs. 20 ± 3 E%, Paleolithic vs. Mediterranean, p = 0.002) (Table 2). The Paleolithic group consumed less carbohydrate in comparisons of both absolute and relative values, and consumed a diet with lower glycemic load and less saturated fatty acids (Table 2). In terms of micronutrients, the Paleolithic group consumed less retinol (but not retinolequivalents), calcium and sodium (Table 2).

**Figure 6:**
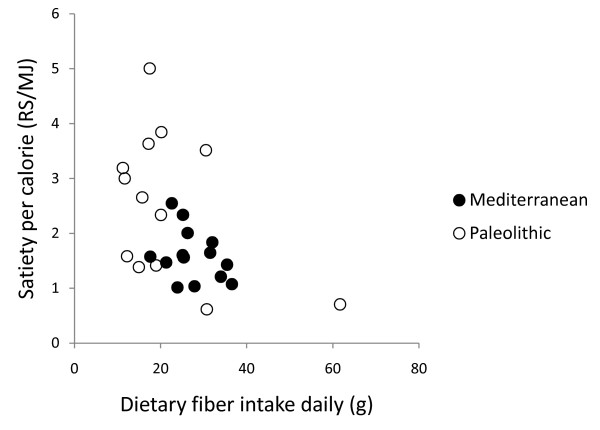
**Individual values of satiety per calorie versus dietary fiber intake daily**. The figure show individual values of quotients of mean change in satiety during meal and mean consumed energy from food and drink versus dietary fiber intake daily. Individuals from the Paleolithic group are depicted with open circles (○) and individuals from the Mediterranean group with closed circles (●).

**Figure 7:**
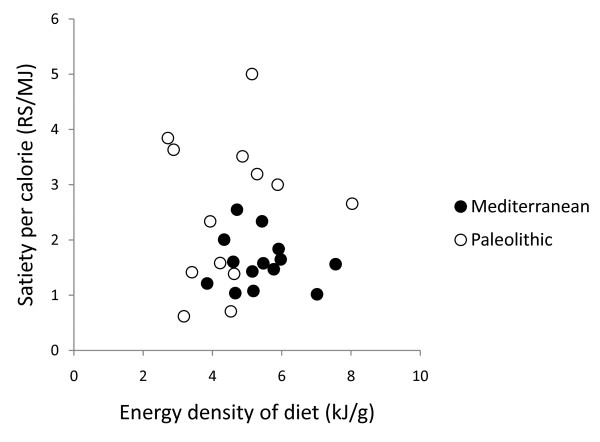
**Individual values of satiety per calorie versus energy density of diet**. The figure show individual values of quotients of mean change in satiety during meal and mean consumed energy from food and drink versus energy density of diet. Individuals from the Paleolithic group are depicted with open circles (○) and individuals from the Mediterranean group with closed circles (●).

**Figure 8:**
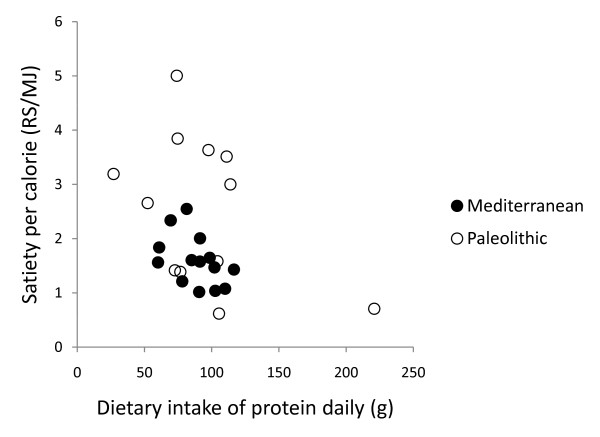
**Individual values of satiety per calorie versus dietary intake of protein daily**. The figure show individual values of quotients of mean change in satiety during meal and mean consumed energy from food and drink versus dietary intake of protein daily. Individuals from the Paleolithic group are depicted with open circles (○) and individuals from the Mediterranean group with closed circles (●).

**Figure 9:**
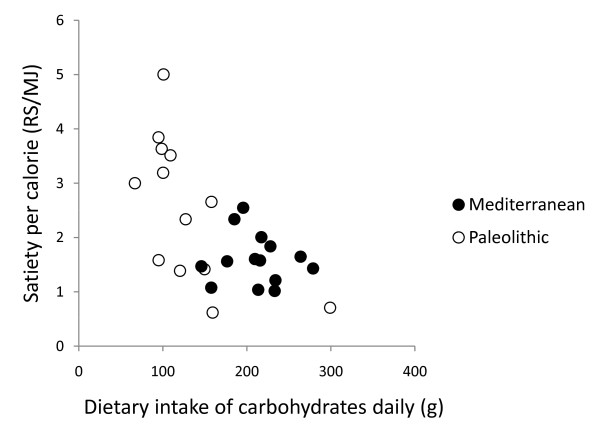
**Individual values of satiety per calorie versus dietary intake of carbohydrates daily**. The figure show individual values of quotients of mean change in satiety during meal and mean consumed energy from food and drink versus dietary intake of carbohydrates daily. Individuals from the Paleolithic group are depicted with open circles (○) and individuals from the Mediterranean group with closed circles (●).

**Figure 10:**
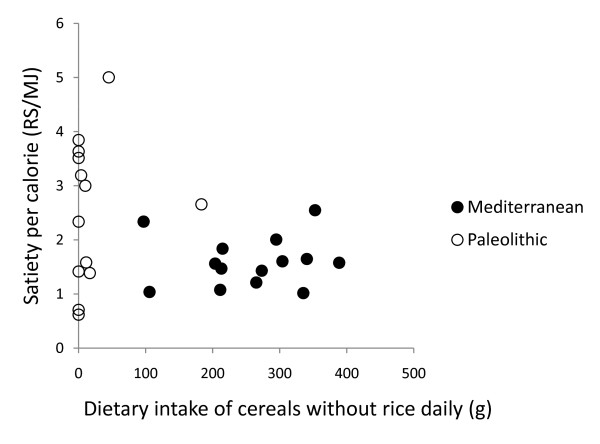
**Individual values of satiety per calorie versus dietary intake of cereals without rice daily**. The figure show individual values of quotients of mean change in satiety during meal and mean consumed energy from food and drink versus dietary intake of cereals without rice daily. Individuals from the Paleolithic group are depicted with open circles (○) and individuals from the Mediterranean group with closed circles (●).

In post hoc analysis, quotients of mean change in satiety during meal and mean consumed energy from food and drink did not correlate with any of the group dietary differences (intake of energy, protein, carbohydrates, GL, saturated fatty acid, fatty acid C14:0, vitamin A, calcium, sodium, fruits, nuts, meat, cereals without rice, rice, milk/milk products) except for fatty acid C4:0-10.0 (Pearson correlation 0.44, p = 0.03) and fatty acid C12 (Pearson correlation 0.43, p = 0.03), and also did not correlate with fiber, energy density, water or beverages (Figure 6, 7, 8, 9 and 10). Among the group dietary differences there was a correlation between Satiety Quotient for energy and intake of energy (Pearson correlation 0.54, p = 0.004), absolute intake of carbohydrates (Pearson correlation 0.50, p = 0.007), GL (Pearson correlation 0.50, p = 0.007), saturated fatty acids (Pearson correlation 0.41, p = 0.03) and sodium (Pearson correlation 0.51, p = 0.007).

## Discussion

### Key findings

After recalculating the nutrient composition reportedly consumed by both groups, we have corroborated our previously reported differences between the groups, including the finding that the individuals in the Paleolithic group consumed less energy compared to the Mediterranean group. We also found that there was no difference in subjectively assessed satiation between the groups. Consequently, the quotients of mean change in satiety during meal and mean consumed energy from food and drink were higher in the Paleolithic group. Also, there was a strong trend for greater Satiety Quotient for energy in the Paleolithic group. Thus, the Paleolithic diet was apparently more satiating per calorie than the Mediterranean. Leptin levels decreased significantly in both groups, with a weak trend for greater relative decrease in the Paleolithic group, which becomes significant if a Paleolithic outlier in terms of cereal intake is excluded. Leptin receptor increased in both groups, and free leptin index decreased in both groups, with no differences between groups. Relative changes in leptin and changes in weight and waist circumference correlated significantly in the Paleolithic group but not in the Mediterranean group. Our previously reported marked improvement of glucose tolerance from this study was not correlated to new data reported here on satiety, leptin, leptin receptor or free leptin index.

### Possible mechanism and explanations

The Paleolithic diet was more satiating per calorie despite no group difference in supposedly satiating fiber intake [34], which also did not correlate with measures of satiety per calorie. This greater satiating capacity may instead have been caused by the trend for lower energy density of the Paleolithic diet [7,35], although energy density did not correlate with measures of satiety per calorie either. Water incorporated into a food increases its satiating capacity through reduced energy density [36], but we found no difference between groups in calculated water content of respective diets or any correlation with measures of satiety per calorie. Differences in beverage intake could also have affected satiety [37], but we found no such differences between the groups or correlation with measures of satiety per calorie. Another possible explanation of the Paleolithic diets greater satiating capacity is the significantly higher relative intake of protein in the Paleolithic group, 27 ± 6% of dietary energy, compared to 20.5 ± 3.6% in the Mediterranean group, which would be consistent with reported reductions in appetite and *ad libitum *caloric intake by high-protein diets [38-40]. However, there was no correlation between relative protein intake and measures of satiety per calorie. Also, since there was no difference in absolute intake of protein, the difference in relative protein intake is probably an effect rather than a cause of the Paleolithic diets greater satiating capacity. Instead, the significantly lower carbohydrate intake in both absolute and relative terms, paired with the greater relative protein intake, could cause the greater satiating capacity of the Paleolithic diet. The Paleolithic diet in this study plays out as a low-carbohydrate diet, and the short-term effects on weight loss from low-carbohydrate diets suggesting greater satiety could be the controlling factor behind the greater satiating effect of the Paleolithic diet in this study [41]. Many studies show that a carbohydrate-restricted diet produce greater short-term (6 months) weight loss than low-fat, calorie-restricted diets, suggesting a greater satiating capacity, although longer-term (1 to 2 years) results are mixed [42]. There was a correlation between the Satiety Quotient for energy and absolute intake of carbohydrate and GL but not for the relative intake of carbohydrates. In a previous long-term study on effects of macronutrients in isocaloric meals on self-reported appetite, Beasley et al found reduced pre-meal appetite from a protein-rich diet compared to a carbohydrate-rich diet [40]. Results from single-meal studies are more ambiguous ranging from no effect on satiety after varying carbohydrate intake from breakfast meals [43,44] to suppressed hunger after a carbohydrate-rich breakfast compared to a fat-rich breakfast [45,46].

Another possible effect of carbohydrates on satiety could be the group difference in type of carbohydrate consumed. The major source of carbohydrate in the Mediterranean group were cereals, which, according to Holt et al [7], are less satiating than fruit, the major source of carbohydrate in the Paleolithic group. However, cereal and fruit intake did not correlate with measures of satiety per calorie. Yet another conceivable cause of the differences in satiating capacity is the significantly lower salt intake in the Paleolithic group, approximately 3.8 gram salt daily, compared to approximately 8.0 gram salt daily in the Mediterranean group (estimated from sodium intake in Table 2), which could affect palatability [47]. There was a correlation between the Satiety Quotient for energy and sodium intake. Also, since bread and milk products are often considered palatable, the much higher intake of these food items in the Mediterranean group could block satiety signals [48]. The relevance of the significantly lower intake of saturated fatty acids in the Paleolithic group in appetite regulation is equivocal [49], although there was a correlation between the Satiety Quotient for energy and intake of saturated fatty acids.

A trend for greater relative decrease of leptin levels in the Paleolithic group could indicate greater increase in leptin sensitivity [19]. This would hypothetically induce effects equivalent to those reported from rats injected with leptin, where energy intake per meal decreased [50], an effect which closely resembles the results from our study. Previous studies indicate that the difference in carbohydrate intake could explain the trend for greater relative decrease of leptin levels in the Paleolithic group [40,44]. In post hoc analysis, the strongest correlation between relative change in leptin and dietary variables was with intake of cereals excluding rice. Rice could be calculated separately from other cereals since rice was reported separately from other cereals by the study participants in the weighed food records. This correlation could indicate that dietary components in cereals cause leptin resistance with ensuing disturbance of appetite regulation, which would explain our observed differences in satiating capacity between diets in this study [4]. The correlation also indicates that there is a qualitative difference between rice and other cereals. Furthermore, our finding that relative changes in leptin and changes in weight and waist circumference correlated significantly in the Paleolithic group but not in the Mediterranean group could indicate a disturbed appetite regulation caused by the Mediterranean diet.

### Comparison with findings from other studies

This is the first study to report effects of a Paleolithic diet on subjective satiety and leptin, leptin receptor and free leptin index. In a recent study on forty-one obese healthy subjects, Hermsdorff et al found that eight weeks on a hypocaloric diet based on a Mediterranean dietary pattern lowered leptin from 27.8 ± 4.1 ng/ml to 23.9 ± 3.6 ng/ml, a 14% reduction, which is slightly lower than the 19% and 18% reduction seen in this study at 6 weeks and 12 weeks on a Mediterranean-like diet [51]. Previously, de Luis et al had reported on a study on 65 obese, non-diabetic out-patients where three months on a lifestyle modification program (Mediterranean hypocaloric diet and exercise) lowered leptin levels around 10-14% [52]. The macronutrient and fatty acid composition of the Paleolithic diet in this study is close to a recent estimate of an East African Paleolithic diet [53]. However, depending on the wide range of possible underlying foraging models in this and previous estimates, the possible ranges for both macronutrient and fatty acid composition for a presumably healthy Paleolithic diet are quite large [53].

### Clinical and research implications

Our findings suggest that a Paleolithic diet is more satiating per calorie than a Mediterranean-like diet. This aspect of a Paleolithic diet is vital to any diet intended to facilitate weight-loss in obese patients and thereby mitigate effects of associated diseases, such as ischemic heart disease and diabetes type 2. Further research into possible mechanisms causing this satiating effect of a Paleolithic diet is clearly warranted.

Total protein intake in g per day did not differ between the diets, but, as a result of the difference in total energy intake, the energy percentage (E%) from dietary protein on the Paleolithic diet (27 E%) exceeded US and European recommendations for people with diabetes (<20 E%) [54,55]. The debatable disadvantage for long-term kidney function [56,57] should be weighed against the benefits of attenuated postprandial glycemia when protein replaces starch or glucose [58].

Calcium intake did not meet recommendations for any of the diets, and it was particularly low in the Paleolithic diet. Recent calcium balance studies indicate that human calcium requirements are lower than previously thought [59], and meta-analyses of randomized controlled trials suggest that the effect of calcium supplementation for bone strength is limited [60,61]. It has been suggested that absorption and excretion of calcium are more important than calcium intake for whole-body calcium balance [62]. In this context, the lower content of calcium-binding phytate and the lower dietary acid load from a Paleolithic diet may hypothetically compensate for the low amount of calcium [63]. Supporting this view are the findings of Frassetto et al, where calcium intake remained unchanged and urine calcium decreased after a Paleolithic diet compared to baseline [64].

As has been discussed, there may be a challenge to implement and adopt the Paleolithic diet on a worldwide scale in subjects with type 2 diabetes. However, this aspect is beyond the objective of this paper and requires more research.

## Conclusions

A Paleolithic diet is more satiating per calorie than a Mediterranean-like diet.

## Competing interests

The authors declare that they have no competing interests and sponsors have had no influence on this report.

## Authors' contributions

TJ participated in the design of the study, participated in statistical analysis, and conceived of and wrote the article. YG participated in the design of the article as well as revising it for important intellectual content. CEA participated in the design of the study and participated in the design of the article as well as revising it for important intellectual content. BA participated in the design of the study, carried out the analysis of leptin and leptin receptor, and participated in the design of the article as well as revising it for important intellectual content. SL conceived of and participated in the design, coordination and execution of the study, participated in statistical analysis, conceived of and participated in the design of the article as well as revising it for important intellectual content. All authors read and approved the final manuscript.
